# Supplementary material for: Identification of Novel Umami Peptides from Yak Bone Collagen and Mechanism Exploration Through In Silico Discovery, Molecular Docking, and Electronic Tongue
Source: Foods. 2025 Nov 26;14(23):4057. doi: 10.3390/foods14234057 (PMC12692289; doi:10.3390/foods14234057)
Supplement: Supplementary file 1 [file foods-14-04057-s001.zip › foods-3969063-supplementary.pdf]

Figure S1

VY

HPLC Analysis Report

Product Name :P102281(VY-2)

Column: 4.6×250mm, SinoChrom ODS-BP

Solvent A A: 0.1% Trifluoroacetic Acid in 100% Acetonitrile

Solvent B B: 0.1% Trifluoroacetic Acid in 100% Water

Gradient:

	A	B
0.0min	2%	98%
25.0min	10%	10%
25.1min	100%	0%
30.0min	Stop	

Volume: 5μl

Wavelength: 220nm

Flow rate: 1.0ml/min

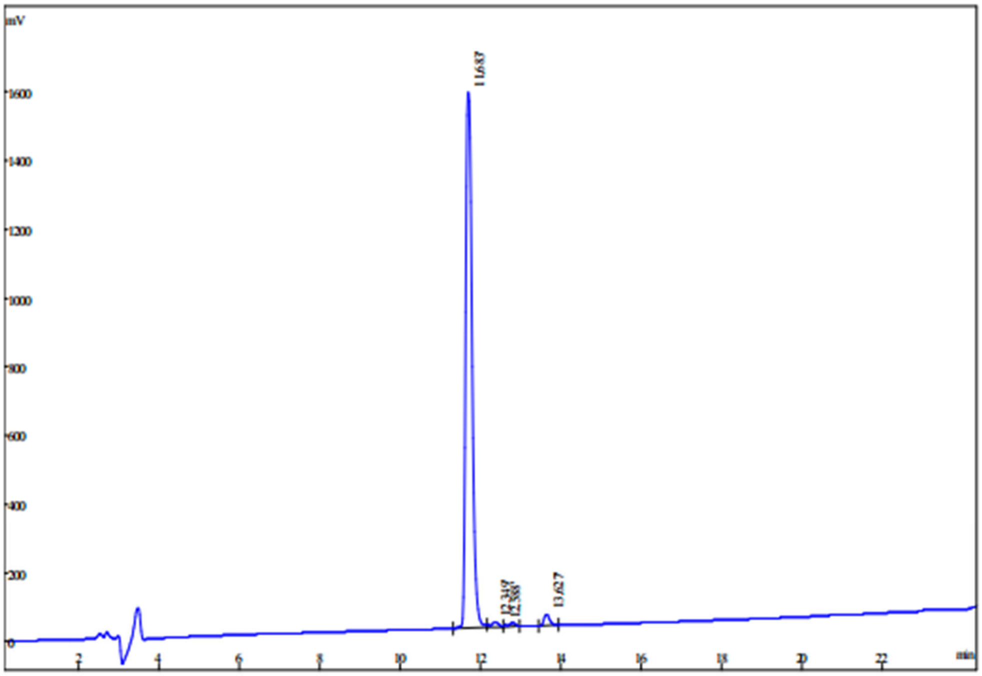

Rank	Time	Conc.	Area	Height
1	11.683	96.41	15531307	1557287
2	12.349	1.05	169217	15159
3	12.588	0.744	119849	3962

4	13.627	1.796	289351	33270
Total		100	16109724	1609678

MS Analysis Report

Product Name:P102281(VY-2)

M.W :280.30

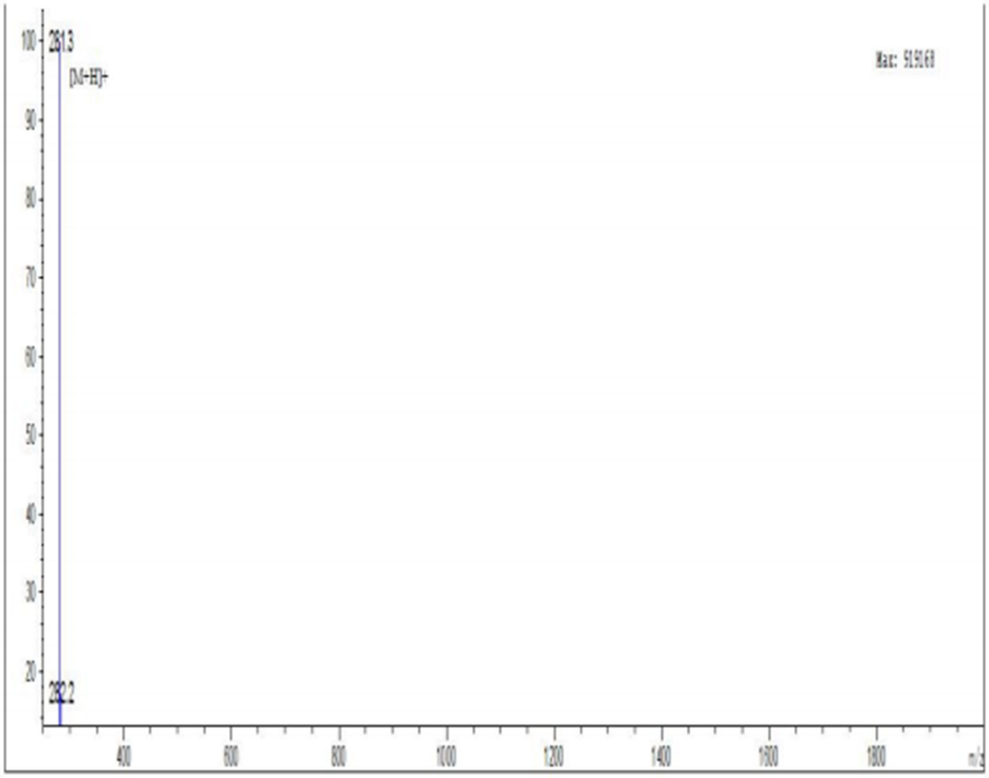

VM

HPLC Analysis Report

Product Name :P102282(VM-2)

Column: Symmetrix ODS-R, 4.6*250mm, 5μm

Solvent A A: 0.1% Trifluoroacetic Acid in 100% Acetonitrile

Solvent B B: 0.1% Trifluoroacetic Acid in 100% Water

	A	B
0.0min	1%	99%
25.0min	26%	74%
25.1min	100%	0%
30.0min	Stop	

Volume: 20μl

Wavelength: 220nm

Flow rate: 1.0ml/min

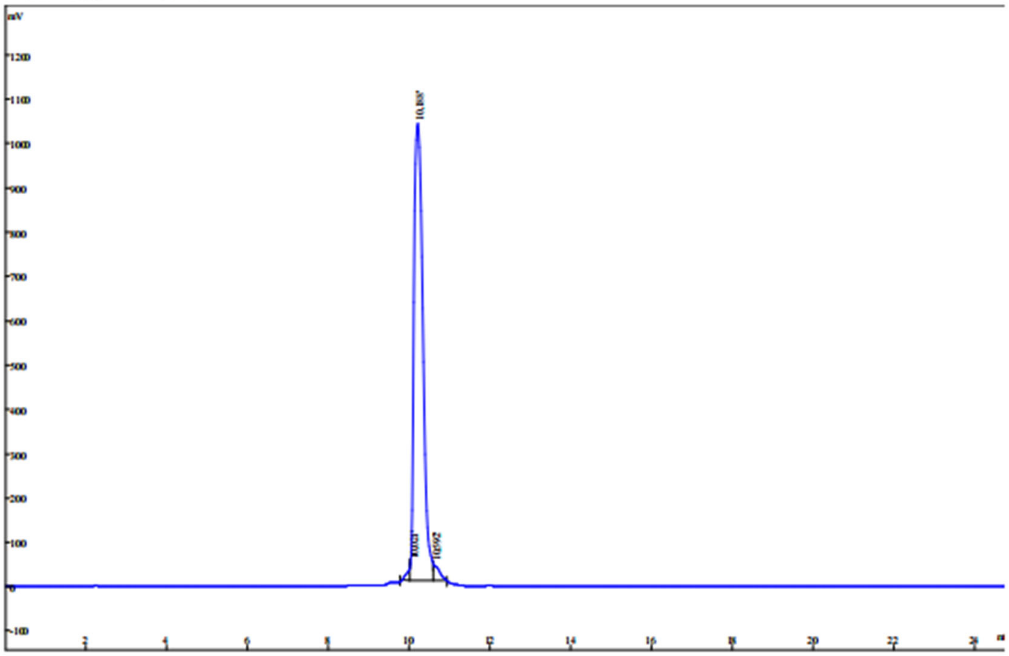

Rank	Time	Conc.	Area	Height
1	10.021	1.269	208895	46392
2	10.188	96.6	15896371	1033396
3	10.592	2.124	349524	35164

Total	100	16454790	1114952

MS Analysis Report

Product Name:P102282(VM-2)

M.W :248.20

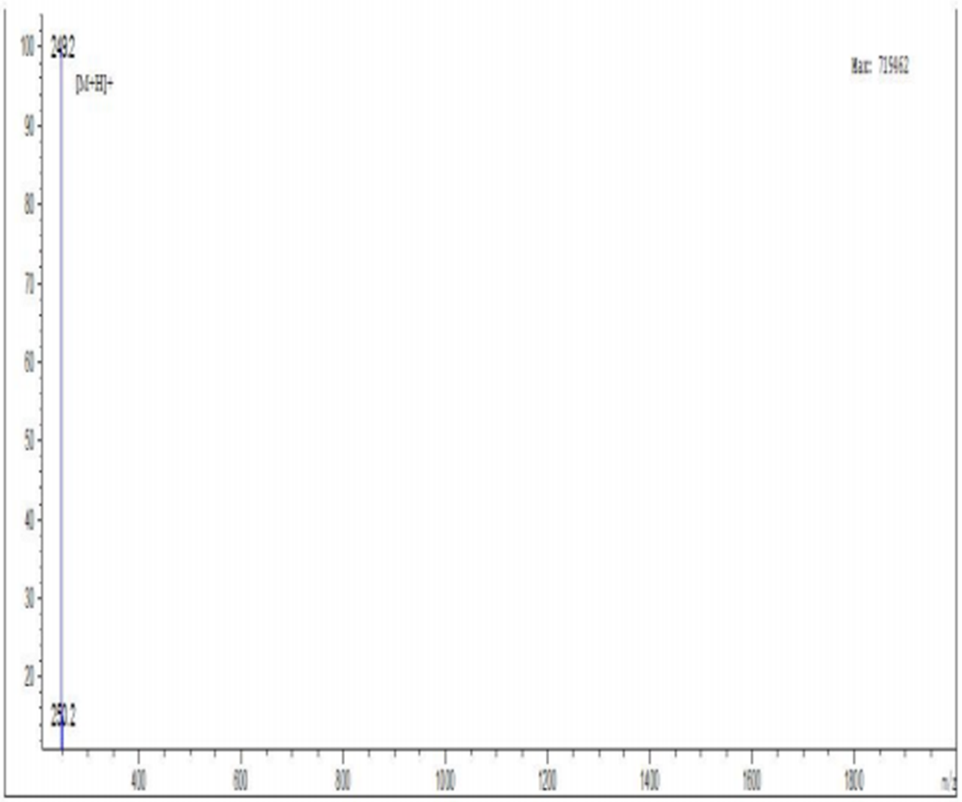

IT

HPLC Analysis Report

Product Name :P102283(IT-2)

Column: 4.6×250mm, SinoChrom ODS-BP

Solvent A A: 0.1% Trifluoroacetic Acid in 100% Acetonitrile

Solvent B B: 0.1% Trifluoroacetic Acid in 100% Water

Gradient:

	A	B
0.0min	2%	98%
25.0min	10%	10%
25.1min	100%	0%
30.0min	Stop	

Volume: 5μl

Wavelength: 220nm

Flow rate: 1.0ml/min

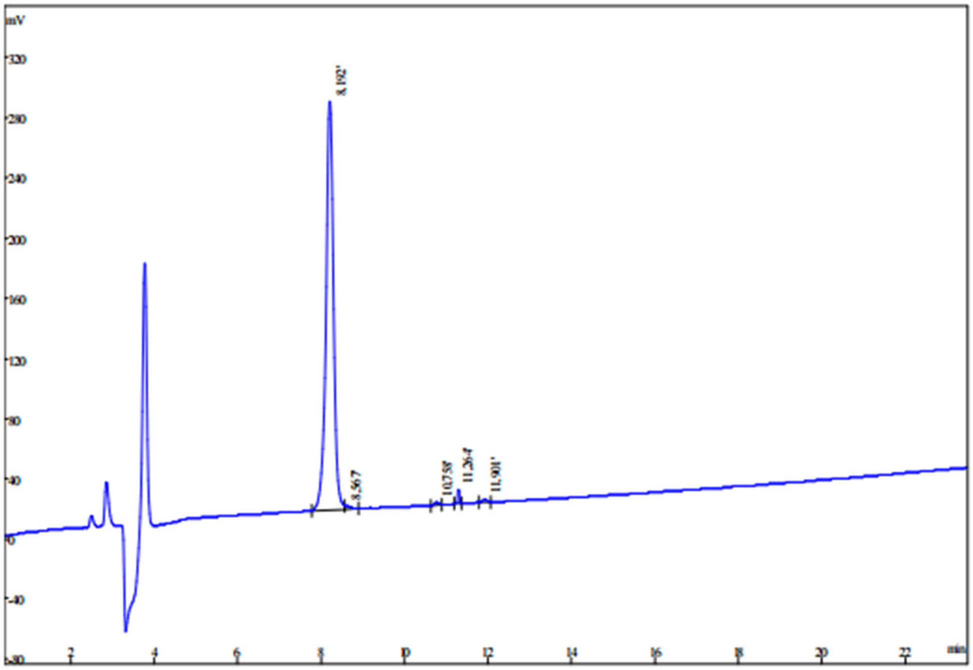

Rank	Time	Conc.	Area	Height
1	8.192	98.11	3125061	271266
2	8.567	0.5929	18885	2405
3	10.758	0.2705	8617	1482

4	11.264	0.6466	20596	9400
5	11.901	0.3795	12089	1899
Total		100	3185248	286452

MS Analysis Report

Product Name:P102283(IT-2)
M.W :232.20

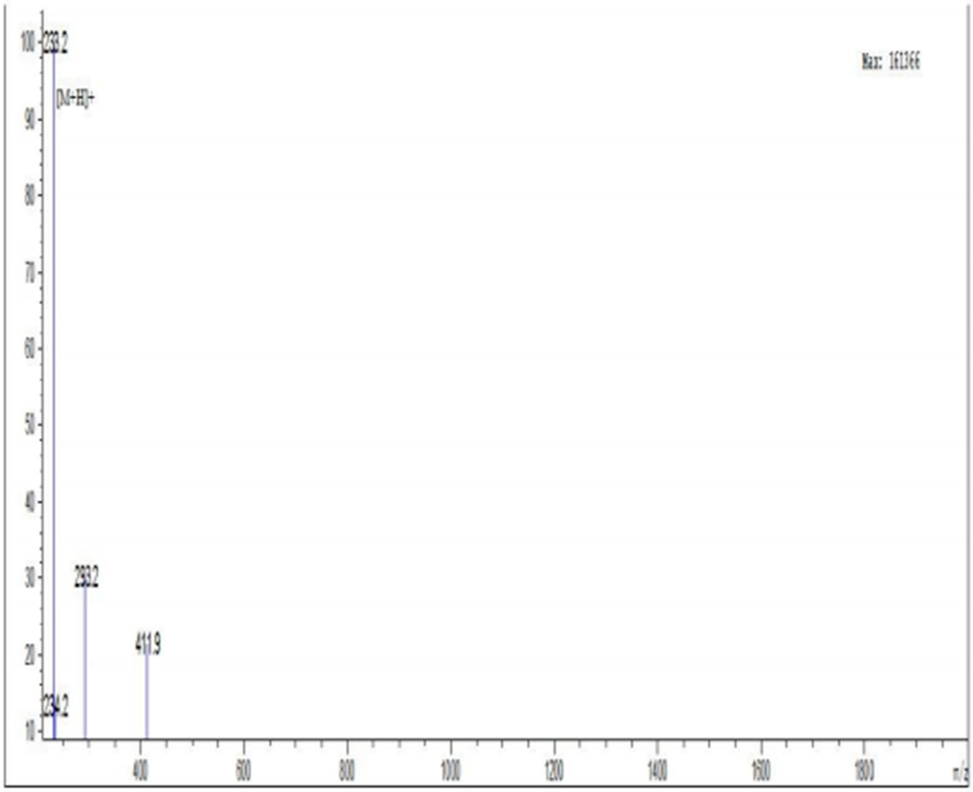

SL

HPLC Analysis Report

**Product Name**	**:P102284(SL-2)**		
**Column:**	**4.6×250mm, SinoChrom ODS-BP**		
**Solvent A**	**A: 0.1% Trifluoroacetic Acid in 100% Acetonitrile**		
**Solvent B**	**B: 0.1% Trifluoroacetic Acid in 100% Water**		
**Gradient:**		**A**	**B**
	**0.0min**	**2%**	**98%**
	**25.0min**	**10%**	**90%**
	**25.1min**	**100%**	**0%**
	**30.0min**	**Stop**	
**Volume:**	**5μl**		
**Wavelength:**	**220nm**		
**Flow rate:**	**1.0ml/min**		

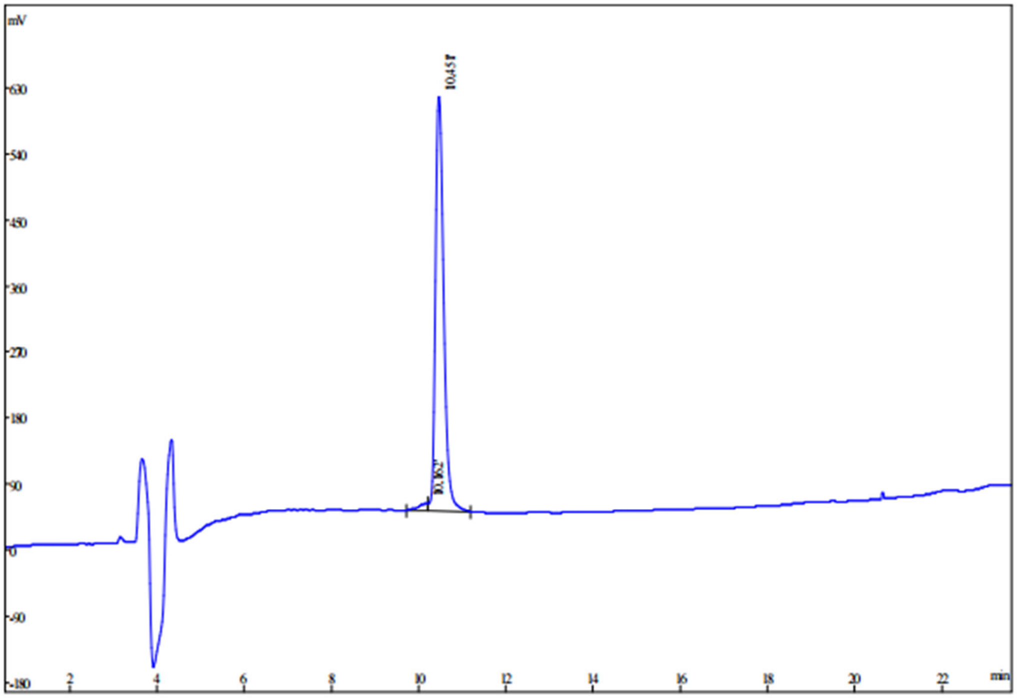

Rank	Time	Conc.	Area	Height
1	10.162	2.195	155438	10149
2	10.451	97.81	6925830	564654

Total 100 7081268 574803

MS Analysis Report

Product Name:P102284(SL-2)

M.W :218.30

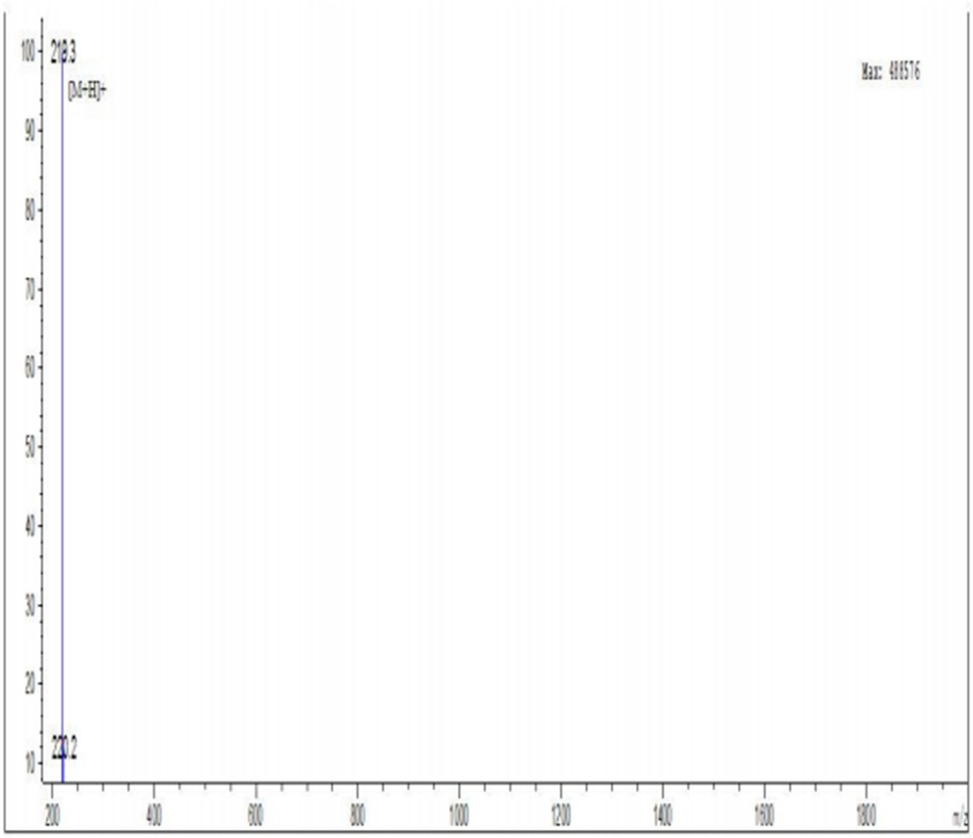

SN

HPLC Analysis Report

Product Name :P102286(SN-2)

Column: 4.6*250mm,GS-120-5-C18-BIO

Solvent A A: 0.1% Trifluoroacetic Acid in 100% Acetonitrile

Solvent B B: 0.1% Trifluoroacetic Acid in 100% Water

Gradient :

	A	B
0.0min	1%	99%
25.0min	1%	99%
25.1min	100%	0%
30.0min	Stop	

Volume: 10µl

Wavelength: 220nm

Flow rate: 0.5ml/min

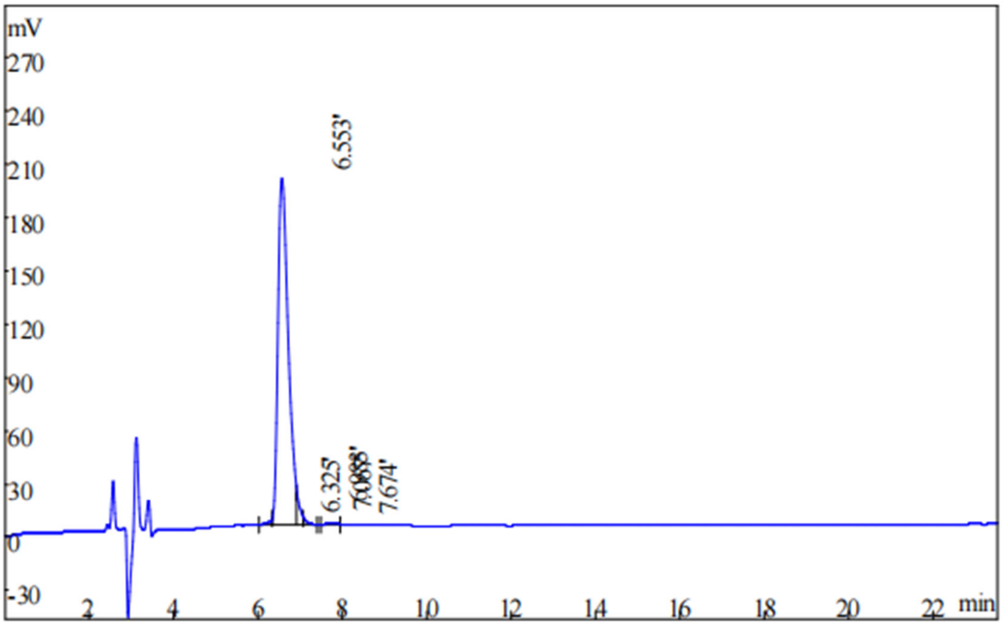

Rank	Time	Quantity	Area	Height
1	6.325	0.5685	18966	2851
2	6.553	95.85	3198121	194446
3	6.983	2.541	84779	8158

4	7.067	0.559	18649	3527
5	7.674	0.4775	15929	1067
**Total**		**100**	**3336444**	**210049**

MS Analysis Report

Product Name:P102286(SN-2)

M.W :219.20

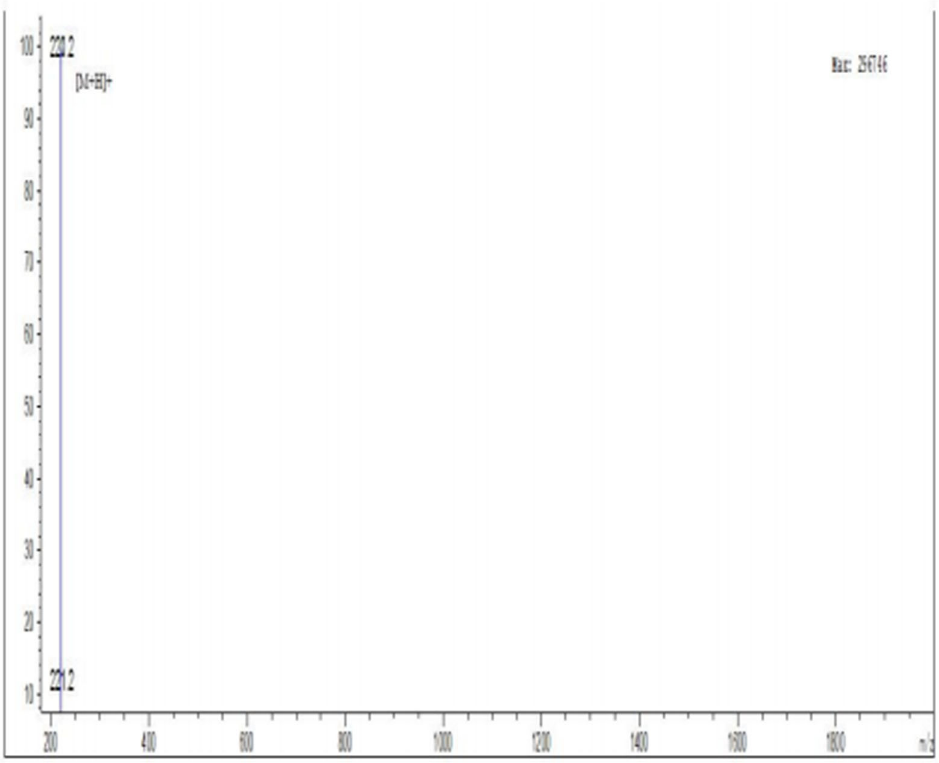

SM

HPLC Analysis Report

**Product Name**	:P102287(SM-2)		
**Column:**	4.6×250mm, SinoChrom ODS-BP		
**Solvent A**	A: 0.1% Trifluoroacetic Acid in 100% Acetonitrile		
**Solvent B**	B: 0.1% Trifluoroacetic Acid in 100% Water		
**Gradient:**		A	B
	0.0min	2%	98%
	25.0min	10%	90%
	25.1min	100%	0%
	30.0min	Stop	
**Volume:**	5µl		
**Wavelength:**	220nm		
**Flow rate:**	1.0ml/min		

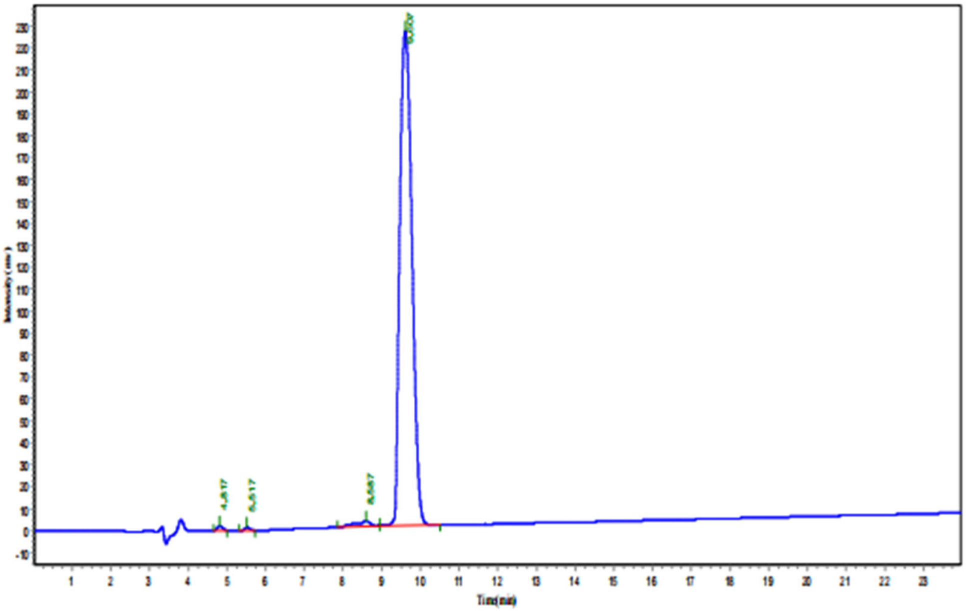

Peak	Time	Height	Area	Conc.
1	4.817	1983.264	19665.100	0.3900
2	5.517	1501.262	13460.000	0.2669
3	8.587	2672.962	79278.320	1.5721
4	9.607	225512.406	4930399.000	97.7710

Total **100.0000**

MS Analysis Report

Product Name:P102287(SM-2)

M.W :236.20

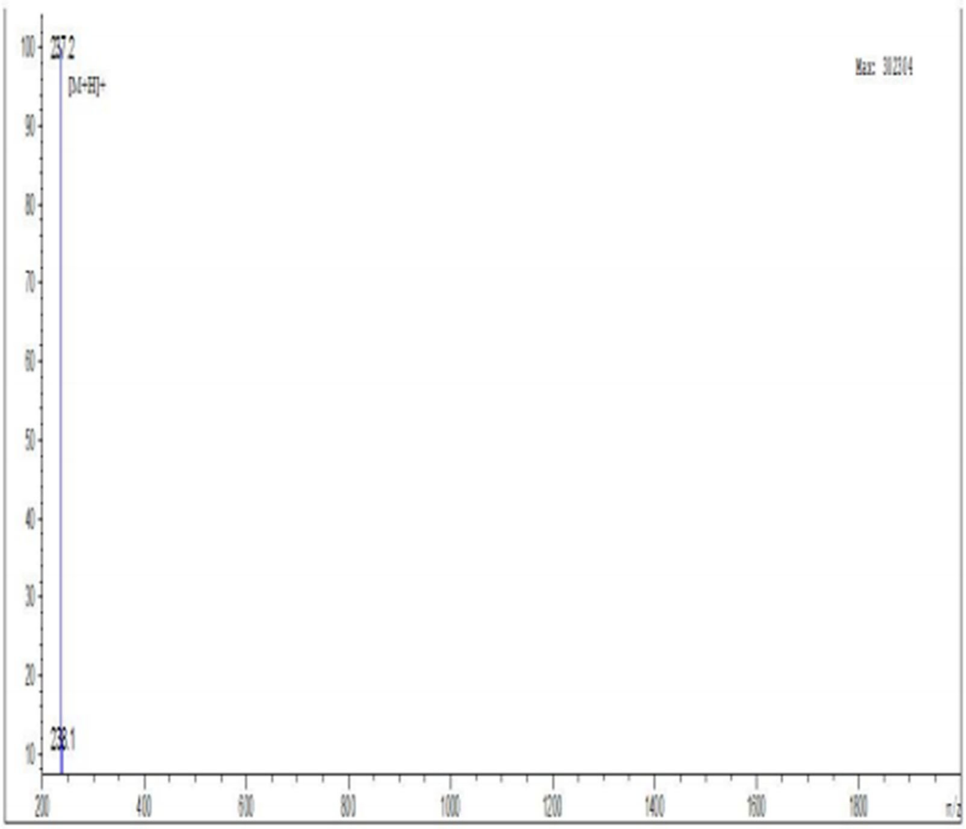

VN

HPLC Analysis Report

Product Name :P102288(VN-2)
Column: 4.6×250mm, SinoChrom ODS-BP
Solvent A A: 0.1% Trifluoroacetic Acid in 100% Acetonitrile
Solvent B B: 0.1% Trifluoroacetic Acid in 100% Water
Gradient:

	A	B
0.0min	2%	98%
25.0min	10%	90%
25.1min	100%	0%
30.0min	Stop	

Volume: 5µl
Wavelength: 220nm
Flow rate: 1.0ml/min

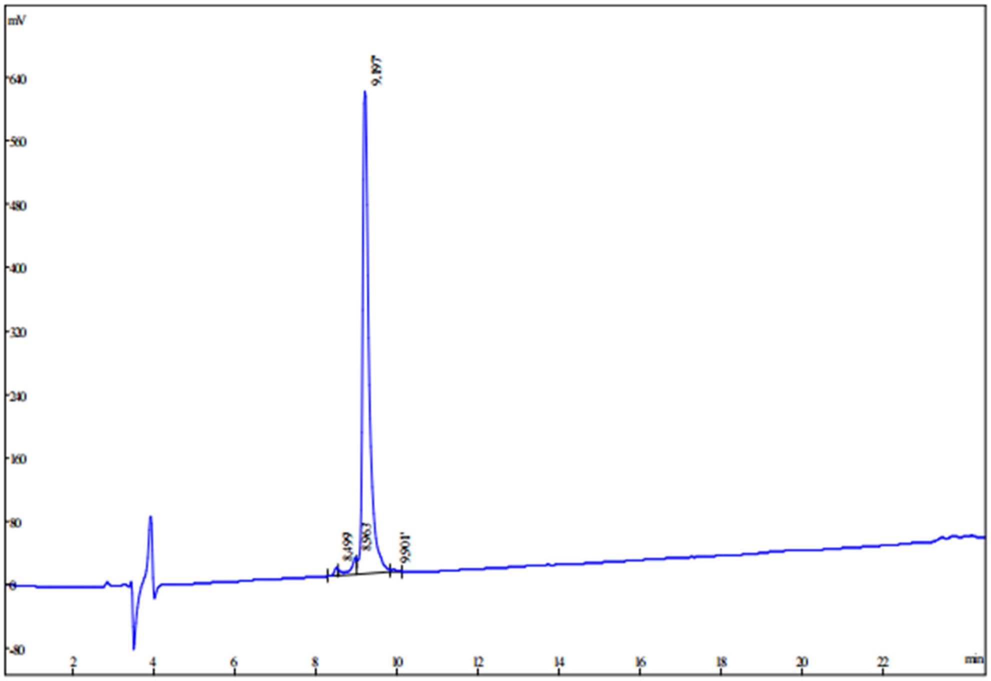

Rank	Time	Conc.	Area	Height
1	8.499	0.9006	57567	8914
2	8.963	3.345	213790	20134
3	9.197	95.32	6093329	607750

4	9.901	0.4321	27618	2845
Total		100	6392304	639643

MS Analysis Report

Product Name:P102288(VN-2)
M.W :231.20

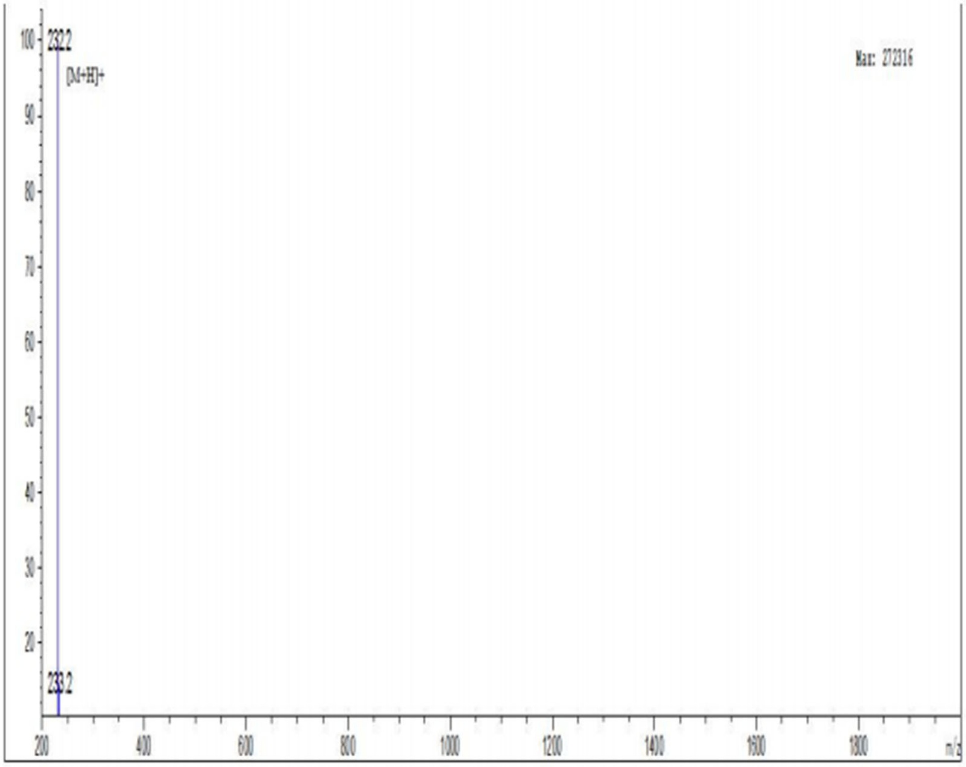

IS

HPLC Analysis Report

**Product Name**	**:P102290(IS-2)**		
**Column:**	**Symmetrix ODS-R, 4.6*250mm, 5µm**		
**Solvent A**	**A: 0.1% Trifluoroacetic Acid in 100% Acetonitrile**		
**Solvent B**	**B: 0.1% Trifluoroacetic Acid in 100% Water**		
		**A**	**B**
	**0.0min**	**1%**	**99%**
	**25.0min**	**26%**	**74%**
	**25.1min**	**100%**	**0%**
	**30.0min**	**Stop**	
**Volume:**	**20µl**		
**Wavelength:**	**220nm**		
**Flow rate:**	**1.0ml/min**		

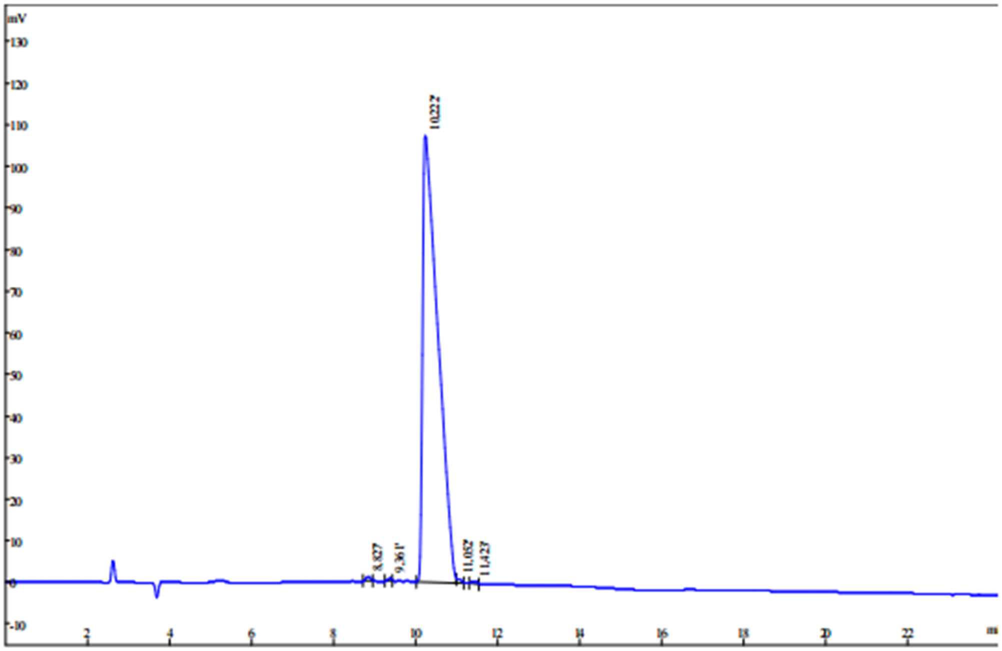

**Rank**	**Time**	**Conc.**	**Area**	**Height**
**1**	**8.827**	**0.2714**	**7289**	**1035**
**2**	**9.361**	**0.1268**	**3406**	**586**
**3**	**10.222**	**99.09**	**2660738**	**107353**
**4**	**11.052**	**0.3062**	**8222**	**1103**

5	11.423	0.2104	5650	782
Total		100	2685305	110859

MS Analysis Report

Product Name:P102290(IS-2)

M.W :218.20

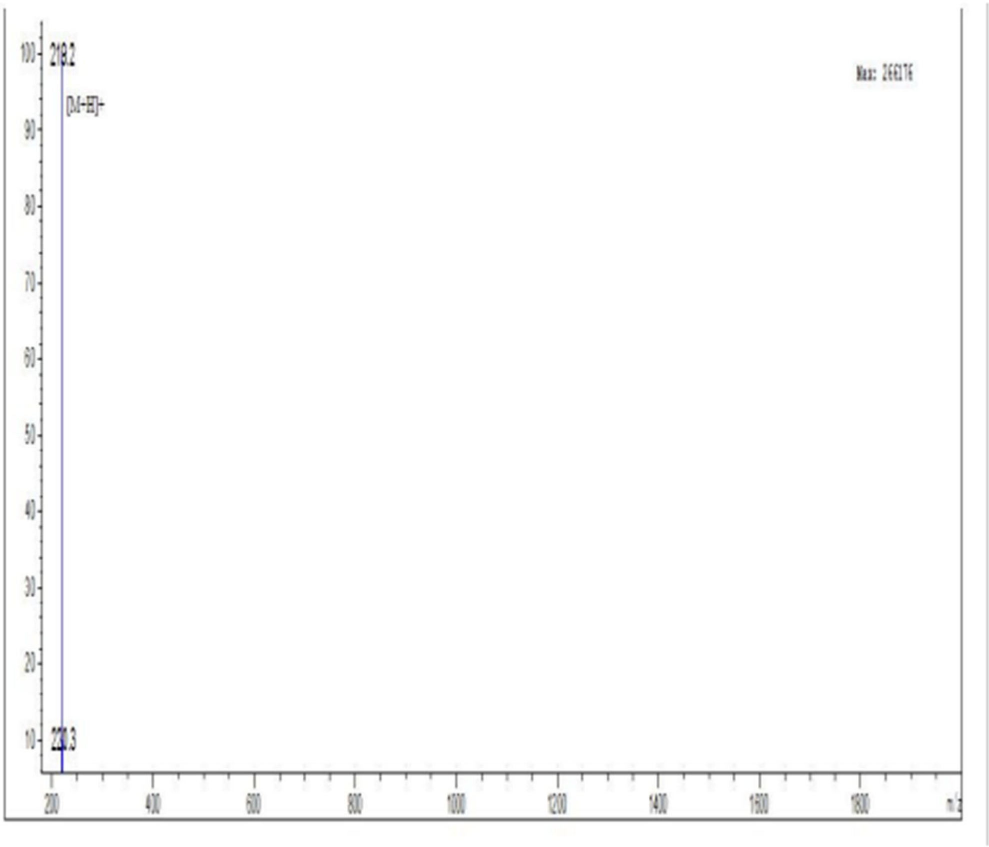

HN

HPLC Analysis Report

**Product Name**	**:P102291(HN-2)**		
**Column:**	**4.6×250mm, SinoChrom ODS-BP**		
**Solvent A**	**A: 0.1% Trifluoroacetic Acid in 100% Acetonitrile**		
**Solvent B**	**B: 0.1% Trifluoroacetic Acid in 100% Water**		
**Gradient:**		**A**	**B**
	**0.0min**	**2%**	**98%**
	**25.0min**	**10%**	**90%**
	**25.1min**	**100%**	**0%**
	**30.0min**	**Stop**	
**Volume:**	**5μl**		
**Wavelength:**	**220nm**		
**Flow rate:**	**1.0ml/min**		

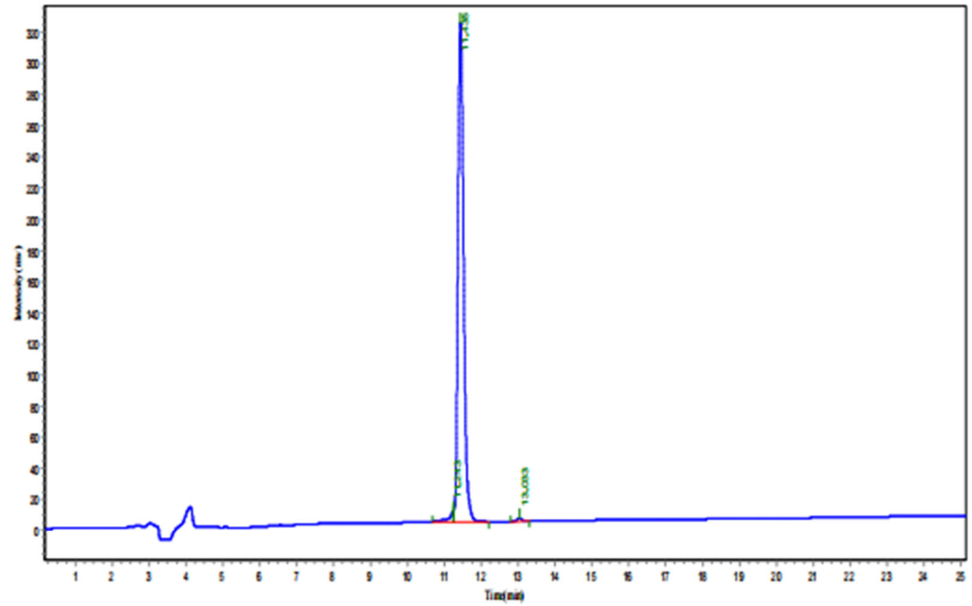

Peak	Time	Height	Area	Conc.
1	11.243	6406.484	50676.402	1.4459
2	11.435	320290.688	3434461.750	97.9919
3	13.033	2126.980	19705.396	0.5622

**Total**	**100.0000**

MS Analysis Report

Product Name:P102291(HN-2)

M.W :269.20

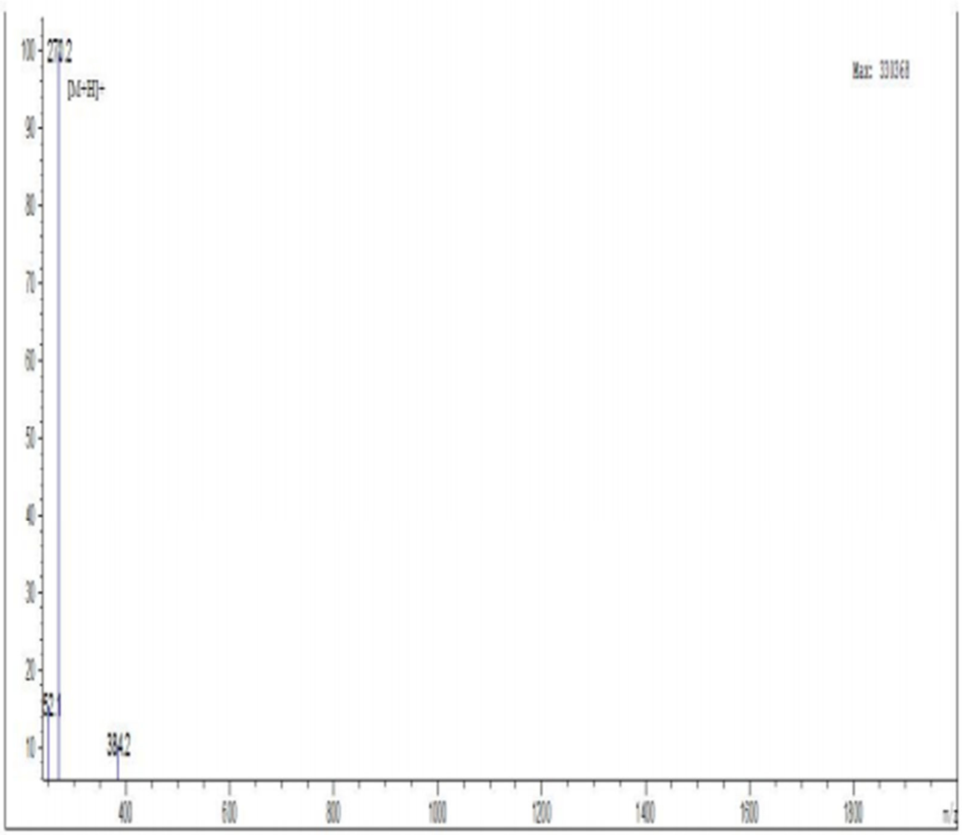

Table S1

Sequence	-CE(kal/mol)	-CIE(kal/mol)
DA	58.6531	39.2982
DG	56.2258	38.0941
HD	43.2784	39.5679
ID	40.4735	44.6891
IE	43.5599	43.9208
PN	32.8198	42.3851
PSG	29.4134	45.0569
SD	48.1987	41.6452
SE	54.4429	45.6965
SY	26.5534	35.2289
VD	40.3299	43.2706
VE	41.6084	36.3654
VG	42.2274	35.9613
VT	44.6798	41.7319
